# Recurrent severe COVID-19 with Good’s syndrome in a patient after thymoma surgery: A case report

**DOI:** 10.1097/MD.0000000000046198

**Published:** 2026-05-12

**Authors:** Hao Zhang, Huan Liu

**Affiliations:** a Department of Respiratory, Shenyang Tenth People’s Hospital, Shenyang Chest Hospital, Shenyang, China.

**Keywords:** case report, COVID-19, Good’s syndrome, immunodeficiency, recurrent infection, thymoma

## Abstract

**Rationale:**

Good’s syndrome (GS) is a rare adult-onset immunodeficiency characterized by the coexistence of thymoma and profound defects in both humoral and cellular immunity. Because of these immune abnormalities, patients are highly vulnerable to recurrent and severe infections, such as COVID-19. However, clinical data on the course and management of COVID-19 in GS remain scarce. This case report aims to describe a patient with recurrent severe COVID-19 following thymectomy and to highlight the diagnostic and therapeutic importance of early immunologic evaluation and intravenous immunoglobulin (IVIG) therapy in this context.

**Patient concerns:**

A 60-year-old man with a history of thymoma resection presented with recurrent episodes of severe COVID-19 pneumonia, each necessitating hospitalization and oxygen therapy. Despite antiviral and corticosteroid treatment, his condition worsened, with persistent hypoxemia and progressive lung infiltrates. Immunologic workup revealed near-complete B-cell depletion and reduced CD4 + T-cell counts, leading to a diagnosis of GS.

**Diagnosis:**

GS following thymoma resection, presenting with recurrent severe COVID-19 complicated by bacterial superinfection.

**Interventions:**

The patient received antiviral agents, corticosteroids, and broad-spectrum antibiotics across multiple hospitalizations. After diagnosis, IVIG therapy was initiated.

**Outcomes:**

IVIG administration led to rapid clinical stabilization and marked radiographic improvement. The patient remained free of relapse during follow-up.

**Lessons:**

This case highlights the importance of early immunologic assessment in patients with atypical or recurrent viral infections post-thymectomy. Prompt recognition of GS and initiation of IVIG therapy can be critical to achieving favorable outcomes.

## 1. Introduction

COVID-19, caused by severe acute respiratory syndrome coronavirus 2 (SARS-CoV-2), has exerted a profound public health impact globally. Although most individuals recover with mild or moderate symptoms,^[1]^ patients with immunosuppression and immunodeficiency carry an increased risk of severe complications.^[2]^ Good’s syndrome (GS) is characterized by thymoma accompanied by immunodeficiency, which results in impaired B- and T-cell function.^[3]^ Approximately 6% to 10% of patients with thymoma develop GS, which predominantly affects individuals between 40 and 60 years of age.^[4]^ In this study, we present a case of recurrent severe COVID-19 in a man with GS following thymoma surgery. The simultaneous occurrence of these 2 entities during a recurrent episode is extremely rare. We have therefore reviewed our experience and provided valuable insights for clinicians managing similar complex conditions. This study was approved by the Research Ethics Committee of the hospital (approval no. KYXM-2024-013-01). The study adhered to the Declaration of Helsinki. The patient provided written informed consent for publication of the case details and accompanying images.

## 2. Case presentation

The patient, a 60-year-old man with a medical history of thymoma, underwent total thymectomy in 2020. Postoperatively, he did not receive any chemotherapy or adjuvant radiotherapy, and no significant comorbidities or recurrent infections had been reported since his surgery. The patient was a nonsmoker, and his family history was unremarkable. He had not received any COVID-19 vaccination prior to this admission or during the subsequent hospitalizations described in this report.

**Figure 1.**
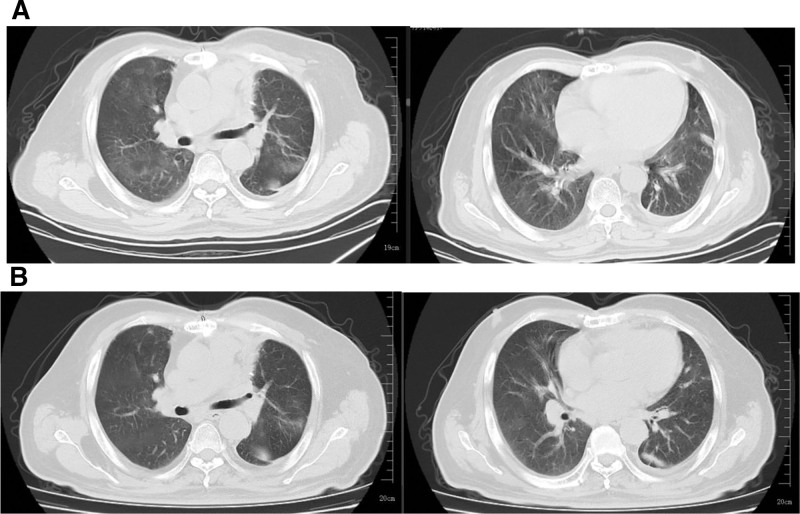
Chest CT images from the January 2023 episode: (A) on admission showing bilateral patchy ground-glass opacities and linear stranding and (B) 1 week post-discharge showing reduced size and fading of opacities. CT: computed tomography.

On January, 2023, the patient was admitted to our hospital because of persistent fever (peak temperature, 38.6°C) and shortness of breath for 2 weeks. He had self-medicated with azvudine tablets (5 mg/daily) for 7 days without any noticeable improvement. Upon admission, the physical examination revealed a respiratory rate of 35 breaths/min, reverse transcription polymerase chain reaction (RT-PCR) using a throat swab was positive for SARS-CoV-2, and chest computed tomography (CT) revealed bilateral patchy ground-glass opacities and fibrous streaks (Fig. 1A). Arterial blood gas analysis revealed a pH of 7.52, partial pressure of oxygen (pO_2_) of 41.2 mm Hg, partial pressure of carbon dioxide (pCO_2_) of 31.2 mm Hg, and a pO_2_/fraction of inspired oxygen ratio of 196 mm Hg. He was diagnosed with severe COVID-19 and type I respiratory failure, according to the Chinese Diagnosis and Treatment Protocol for Novel Coronavirus Pneumonia (10th edition).^[5]^ Comprehensive treatment was administered to manage his symptoms, which included intravenous dexamethasone (5 mg/daily), antiviral therapy with Xiyanping injection (250 mg/d),^[6]^ and enoxaparin sodium injection (4000 units/d), for 1 week. Following treatment, his symptoms significantly improved, and he was subsequently discharged. One week later, follow-up chest CT (Fig. 1B) revealed reductions in the extent and density of the bilateral patchy opacities, indicating marked clinical improvement.

On July, 2023, the patient developed fever with a peak temperature of 38.5°C, accompanied by chest tightness and shortness of breath. At a local hospital, RT-PCR using a throat swab confirmed the diagnosis of COVID-19, and chest CT revealed ground-glass exudative opacities and patchy lesions in the left lung. During hospitalization, he received levofloxacin (0.5 g/d), Xuebijing injection (50 mL, twice/d),^[7]^ and methylprednisolone sodium succinate injection (40 mg/d) intravenously for 7 days. His fever subsided, and he was subsequently discharged. However, 3 weeks after discharge, the patient again developed fever with a peak temperature of 39.0°C, accompanied by dyspnea, cough, and production of yellow sputum. On August, 2023, he was admitted to our hospital for further evaluation and management.

Upon admission, physical examination revealed the following findings: temperature of 36.8°C, heart rate of 98 beats/min, respiratory rate of 34 breaths/min, and blood pressure of 130/60 mm Hg. The patient was alert and oriented, but he exhibited signs of respiratory distress, including tachypnea and cyanosis of the lips. Auscultation revealed bilateral dry and wet rales. Laboratory investigations demonstrated an elevated white blood cell count of 11.21 × 10^9^/L, a neutrophil percentage of 80.8%, a lymphocyte count of 1.41 × 10^9^/L, and a significantly increased high-sensitivity C-reactive protein level of 147.78 mg/L. Arterial blood gas analysis (with oxygen supplementation at 3 L/min) revealed a pH of 7.50, pO_2_ of 68 mm Hg, pCO_2_ of 29 mm Hg, and a pO_2_/fraction of inspired oxygen ratio of 206 mm Hg. Flow cytometry indicated a CD4 + cell count of 286/μL (ref, 500–1500/μL), CD8 + cell count of 819/μL (ref, 190–1940/μL), CD4+/CD8 + ratio of 0.35 (ref, 0.98–2.50), and B-cell count of 1/μL (ref, 80–616/μL).

**Figure 2.**
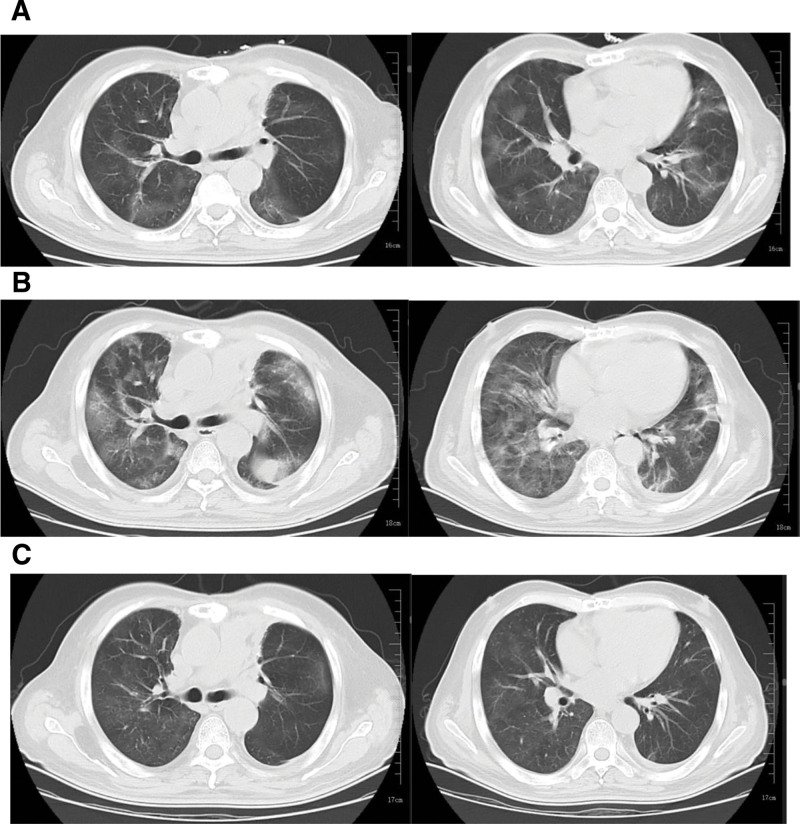
Chest CT images from the July to August 2023 episode: (A) On admission showing consolidation near the mediastinum and scattered ground-glass opacities; (B) during hospitalization showing increased density of patchy shadows; and (C) follow-up in September 2023 showing significant resolution of inflammation.

According to the chest CT findings (Fig. 2A, on admission), bronchoscopy was indicated, and metagenomics next-generation sequencing of bronchoalveolar lavage fluid suggested infections by SARS-CoV-2 (4.85 × 10⁷ copies/mL), *Streptococcus pneumoniae* (2.71 × 10⁵ copies/mL), and *Haemophilus influenzae* (5.44 × 10⁴ copies/mL). Routine bacterial cultures and real-time RT-PCR were not performed. Based on these findings, a diagnosis of severe COVID-19 complicated by bacterial pneumonia was established. The patient was then treated with piperacillin–sulbactam (IV, 5.0 g/12 h), Xiyanping injection (IV, 250 mg/daily), dexamethasone (5 mg/daily), and oral nirmatrelvir (300 mg/12 h) plus ritonavir (100 mg/12 h) for 5 days to provide supportive and symptomatic treatment. Despite these interventions, the patient continued to experience dyspnea, poor oxygenation, and persistent fever. Repeated chest CT revealed worsening density of some patchy opacities bilaterally (Fig. 2B), indicating an inadequate response to treatment.

The patient was subsequently diagnosed with GS after thymoma resection. Despite antibacterial, antiviral, and corticosteroid therapy initiated at admission, he continued to experience dyspnea, hypoxemia, and persistent fever after 5 days, with chest CT showing progression of patchy infiltrates. Immunologic testing revealed marked hypogammaglobulinemia (IgA 0.07 g/L, IgG 2.68 g/L, and IgM 0.04 g/L). Accordingly, intravenous immunoglobulin (IVIG) was initiated on hospital day 6 at a dose of 0.4 g/kg/d for 5 consecutive days, resulting in substantial clinical improvement. On September, 2023, follow-up chest CT highlighted marked resolution of pulmonary inflammation (Fig. 2C), and the patient was subsequently discharged after recovering. As he resided outside the local region and did not return for further evaluation, long-term follow-up data on post-discharge relapse were unavailable.

## 3. Discussion

GS is a rare, acquired immunodeficiency characterized by thymoma-associated hypogammaglobulinemia and profound impairments in both cellular and humoral immunity. These defects predispose individuals to recurrent and often severe infections, particularly those involving the respiratory tract.^[8]^ In this study, the patient underwent thymoma resection. The patient presented with markedly low CD4 + T-cell counts, an inverted CD4+/CD8 + ratio, and an undetectable B-cell count, which are key features of GS, significantly compromising his ability to combat SARS-CoV-2 infection. Previous studies found that thymoma resection does not restore immune function,^[9]^ and patients with immunodeficiency are at high risk for severe COVID-19,^[10,11]^ which might explain the recurrent episodes of severe COVID-19 observed in this patient. Several case reports have described COVID-19 in patients with GS, offering insights into shared patterns and management considerations. Wee et al reported 2 GS patients with relapsing COVID-19, identifying relapsing infection as a clinical manifestation of GS and underscoring challenges in viral clearance in the setting of combined B- and T-cell defects.^[12]^ Berzenji et al described a 55-year-old man with GS who developed COVID-19 during thymoma treatment;^[13]^ he received remdesivir and thymectomy, then continued on 4-weekly immunoglobulin replacement, with prolonged viral shedding and absent serologic response after vaccination but no new infections once on regular IVIG. Compared with previously reported cases, our patient was unvaccinated and experienced 2 severe episodes more than 6 months apart, supporting reinfection rather than relapse. During the second episode, the short-interval recurrence following suboptimal antiviral coverage and the prompt clinical and radiologic improvement after IVIG initiation underscored the contribution of combined humoral and cellular immune defects to disease severity. These findings highlight the importance of early immune evaluation and timely IVIG in the management of COVID-19 in patients with GS.

Although viral genomic sequencing was not performed, the 6-month interval between infections, along with initial clinical recovery, supports reinfection rather than prolonged viral shedding. The pronounced severity of the first episode may have been an early indicator of underlying immune dysfunction, and the lack of immune profiling at that stage likely delayed the diagnosis of GS. This case highlights the importance of targeted immune evaluation in COVID-19 patients with predisposing histories, such as prior thymectomy, who present with unexpectedly severe or recurrent infections, even in the absence of other typical risk factors.

B-cell depletion is a defining feature of GS, resulting in hypogammaglobulinemia and impaired antibody-mediated immunity. Although GS is rare, it is a well-recognized sequela among patients with thymoma or those who have undergone thymectomy. In hindsight, early immunologic assessment, including serum immunoglobulin quantification and lymphocyte subset analysis, should have been conducted during the patient’s initial hospitalization. The omission of such testing delayed both diagnosis and initiation of immune-directed therapy. This case emphasizes the need to consider combined B- and T-cell immunodeficiency in post-thymectomy patients presenting with severe or atypical viral infections. Early immune profiling may expedite diagnosis and facilitate timely interventions.

Profound B-cell depletion (1/μL), a pathognomonic hallmark of GS, resulted in hypogammaglobulinemia and markedly impaired primary and memory antibody responses. In the context of SARS-CoV-2 infection, this deficit precluded the generation of adequate neutralizing antibodies, leaving the patient highly susceptible to reinfection and severe disease. During both the January and July 2023 episodes, the patient did not receive guideline-recommended antiviral agents, such as remdesivir or nirmatrelvir/ritonavir, despite evidence supporting their use in high-risk populations.^[14–16]^ This omission reflected the absence of a recognized immunodeficiency at the time, local constraints in drug availability, and clinical judgment based on disease severity, and may have contributed to incomplete viral clearance. Nonetheless, the patient’s underlying GS was ultimately responsible for impaired viral control.

IVIG therapy proved pivotal in stabilizing the patient’s condition by providing passive immunity through exogenous antibodies that compensated for hypogammaglobulinemia. Following IVIG initiation, the patient experienced rapid clinical and radiological improvement, consistent with current recommendations that emphasize regular immunoglobulin replacement to prevent severe infections in GS.^[17]^ Although randomized data regarding IVIG use in COVID-19 remain limited, accumulating clinical evidence – including case reports and small series – supports its benefit in patients with profound B-cell deficiency.^[18–20]^ Moreover, commercial IVIG preparations increasingly contain anti-SARS-CoV-2 neutralizing antibodies, reflecting widespread donor vaccination and prior viral exposure.^[21]^ This evolving antibody repertoire enhances the therapeutic potential of IVIG, particularly as the efficacy of monoclonal antibodies declines with viral antigenic drift. In this immunologically vulnerable patient, IVIG served as a critical adjunctive therapy, providing both passive immune support and broad-spectrum antiviral activity.

The significant reduction in CD4 + T-cell counts further compromised the cellular immune response, which is essential for effective viral clearance. Data indicate that, compared with healthy individuals, patients with GS who have undergone thymoma resection exhibit an increased CD4+/CD8 + T-cell ratio along with a marked reduction in overall T-cell counts in peripheral blood.^[22]^ Effective clearance of SARS-CoV-2 relies heavily on the coordinated actions of CD4 + and CD8 + T-cell subsets. Notably, a reduction in CD4 + T-cell counts has been linked to prolonged viral persistence in the body.^[23]^ Pro-inflammatory cytokines can establish an inflammatory response loop through monocytes, macrophages, and T lymphocytes, contributing to viral recurrence after apparent recovery from COVID-19. Additionally, SARS-CoV-2 itself exhibits lymphotropic properties, leading to direct damage to the immune system.^[24]^ The virus can affect the cytoplasmic components of lymphocytes, resulting in necrosis or apoptosis of T-cell subsets.^[25,26]^ The reduction in both the quantity and function of T-cell subsets exacerbates immune-mediated interstitial lung disease, thereby increasing the severity of COVID-19 pneumonia. Because of these factors, the patient’s cellular immunity was severely compromised, leading to delayed viral clearance and the progression to severe disease.

This study has several limitations. First, it represents a single-case report, which restricts the ability to generalize the findings to all patients with GS. Second, viral genomic sequencing was not performed, precluding definitive differentiation between reinfection and prolonged viral persistence. Third, long-term follow-up data were unavailable because the patient resided outside the study area. Finally, certain laboratory and immunologic tests could not be repeated during the first hospitalization, which may have delayed diagnostic confirmation. Despite these limitations, this case underscores the clinical importance of early immune evaluation and timely IVIG administration in patients with unexplained recurrent or severe viral infections after thymectomy.

## 4. Conclusion

This rare case underscores the complex interplay among GS, immune dysfunction, and susceptibility to severe COVID-19. Effective management of such patients requires both adherence to standard COVID-19 treatment protocols and the integration of targeted immunological interventions, such as regular IVIG therapy, to address the underlying immunodeficiencies. The observed stabilization of the patient following IVIG therapy highlights the critical role of maintaining adequate immune support to prevent severe recurrent infections. Moreover, this case highlights the importance of long-term follow-up for patients with GS, involving regular immune system evaluations to facilitate timely intervention for recurrent infections or other complications.

## Author contributions

**Writing – original draft**: Hao Zhang, Huan Liu

## References

[1] Zeng N Zhao YM Yan W. A systematic review and meta-analysis of long term physical and mental sequelae of COVID-19 pandemic: call for research priority and action. Mol Psychiatry. 2023;28:423–33. 35668159 10.1038/s41380-022-01614-7 PMC9168643

[2] Gao Y Chen Y Liu M Shi S Tian J. Impacts of immunosuppression and immunodeficiency on COVID-19: a systematic review and meta-analysis. J Infect. 2020;81:e93–5. 10.1016/j.jinf.2020.05.017 PMC7228685 32417309

[3] Sipos F Muzes G. Good’s syndrome: brief overview of an enigmatic immune deficiency. APMIS. 2023;131:698–704. 37729389 10.1111/apm.13351

[4] Guevara-Hoyer K Fuentes-Antras J Calatayud Gastardi J Sanchez-Ramon S. Immunodeficiency and thymoma in Good syndrome: two sides of the same coin. Immunol Lett. 2021;231:11–7. 33418010 10.1016/j.imlet.2020.12.010

[5] China RbNHCoPsRo, National Administration of Traditional Chinese Medicine on January 5. Diagnosis and treatment protocol for COVID‐19 patients (Tentative 10th Version). Health Care Sci. 2023;2:10–24. 38939741 10.1002/hcs2.36 PMC11080886

[6] Wang ZF Zhang HC Xie YM. Expert consensus statement on Xiyanping Injection for respiratory system infectious diseases in clinical practice(adults). Zhongguo Zhong Yao Za Zhi. 2019;44:5282–6. 32237369 10.19540/j.cnki.cjcmm.20191105.502

[7] Chen M Shu W Zhang J Huang H Liu J. Mechanisms and clinical application of Xuebijing injection, a traditional Chinese herbal medicine–a systematic review. Adv Tradit Med. 2024;24:403–12.

[8] Kabir A Polito V Tsoukas CM. Unraveling the natural history of Good’s syndrome: a progressive adult combined immunodeficiency. J Allergy Clin Immunol Pract. 2024;12:744–52.e3. 38122866 10.1016/j.jaip.2023.12.018

[9] Roosen J Oosterlinck W Meyns B. Routine thymectomy in congenital cardiac surgery changes adaptive immunity without clinical relevance. Interact Cardiovasc Thorac Surg. 2015;20:101–6. 25320142 10.1093/icvts/ivu343

[10] Treskova-Schwarzbach M Haas L Reda S. Pre-existing health conditions and severe COVID-19 outcomes: an umbrella review approach and meta-analysis of global evidence. BMC Med. 2021;19:212. 34446016 10.1186/s12916-021-02058-6 PMC8390115

[11] Wang Y Xie Y Hu S. Systematic review and meta-analyses of the interaction between HIV infection and COVID-19: two years’ evidence summary. Front Immunol. 2022;13:864838. 35619709 10.3389/fimmu.2022.864838 PMC9128408

[12] Wee LE Tan JY Oon LLE. Relapsing COVID-19 infection as a manifestation of Good syndrome: a case report and literature review. Int J Infect Dis. 2023;129:236–9. 36608786 10.1016/j.ijid.2022.12.040 PMC9809144

[13] Berzenji L Yogeswaran SK Snoeckx A Van Schil PE Wener R Hendriks JMH. Good’s syndrome and COVID-19: case report and literature review. Mediastinum. 2023;7:5. 36926289 10.21037/med-22-12 PMC10011861

[14] Wildner NH Ahmadi P Schulte S. B cell analysis in SARS-CoV-2 versus malaria: increased frequencies of plasmablasts and atypical memory B cells in COVID-19. J Leukoc Biol. 2021;109:77–90. 33617048 10.1002/JLB.5COVA0620-370RR PMC10016889

[15] Woodruff MC Ramonell RP Nguyen DC. Extrafollicular B cell responses correlate with neutralizing antibodies and morbidity in COVID-19. Nat Immunol. 2020;21:1506–16. 33028979 10.1038/s41590-020-00814-z PMC7739702

[16] Rajamanickam A Kumar NP Nancy PA. Recovery of memory B-cell subsets and persistence of antibodies in convalescent COVID-19 patients. Am J Trop Med Hyg. 2021;105:1255–60. 34583334 10.4269/ajtmh.21-0883 PMC8592221

[17] Orange JS Hossny EM Weiler CR. Use of intravenous immunoglobulin in human disease: a review of evidence by members of the Primary Immunodeficiency Committee of the American Academy of Allergy, Asthma and Immunology. J Allergy Clin Immunol. 2006;117:S525–53. 16580469 10.1016/j.jaci.2006.01.015

[18] Groning R Walde J Ahlm C Forsell MNE Normark J Rasmuson J. Intravenous immunoglobulin therapy for COVID-19 in immunocompromised patients: a retrospective cohort study. Int J Infect Dis. 2024;144:107046. 38615825 10.1016/j.ijid.2024.107046

[19] Maruki T Nomoto H Iwamoto N. Successful management of persistent COVID-19 using combination antiviral therapy (nirmatrelvir/ritonavir and remdesivir) and intravenous immunoglobulin transfusion in an immunocompromised host who had received CD20 depleting therapy for follicular lymphoma. J Infect Chemother. 2024;30:793–5. 38242284 10.1016/j.jiac.2024.01.008

[20] Billi B Cholley P Grobost V. Intravenous immunoglobulins for the treatment of prolonged COVID-19 in immunocompromised patients: a brief report. Front Immunol. 2024;15:1399180. 38707896 10.3389/fimmu.2024.1399180 PMC11069322

[21] Upasani V Townsend K Wu MY. Commercial immunoglobulin products contain neutralizing antibodies against severe acute respiratory syndrome coronavirus 2 spike protein. Clin Infect Dis. 2023;77:950–60. 37338118 10.1093/cid/ciad368 PMC10552578

[22] Chen X Zhang JX Shang WW Xie WP Jin SX Wang F. Aberrant peripheral immune function in a Good syndrome patient. J Immunol Res. 2018;2018:6212410. 29850635 10.1155/2018/6212410 PMC5937423

[23] Ling Y Xu SB Lin YX. Persistence and clearance of viral RNA in 2019 novel coronavirus disease rehabilitation patients. Chin Med J (Engl). 2020;133:1039–43. 32118639 10.1097/CM9.0000000000000774 PMC7147278

[24] Peng X Ouyang J Isnard S. Sharing CD4+ T cell loss: when COVID-19 and HIV collide on immune system. Front Immunol. 2020;11:596631. 33384690 10.3389/fimmu.2020.596631 PMC7770166

[25] Cizmecioglu A Akay Cizmecioglu H Goktepe MH. Apoptosis-induced T-cell lymphopenia is related to COVID-19 severity. J Med Virol. 2021;93:2867–74. 33331657 10.1002/jmv.26742

[26] Donia A Bokhari H. Apoptosis induced by SARS-CoV-2: can we target it? Apoptosis. 2021;26:7–8. 33512610 10.1007/s10495-021-01656-2 PMC7844099

